# Retraction: Livestock Drugs and Disease: The Fatal Combination behind Breeding Failure in Endangered Bearded Vultures

**DOI:** 10.1371/annotation/1a7d5b6c-fde5-4d60-a6df-0e98f754c376

**Published:** 2012-10-31

**Authors:** Guillermo Blanco, Jesús A. Lemus

Dr. Blanco, the first author, and the editors retract this publication.

The Ethics Committee of the Spanish Superior Council of Scientific Research (CSIC) has carried out a formal investigation in relation to concerns about potential scientific misconduct by Jesús A. Lemus, the second author of this article. The investigation has questioned the validity of the laboratory analyses conducted by Dr. Lemus.

Given the ephemeral nature of the material used (liver, bursa of Fabricius, etc.), it is not possible to repeat these analyses with the same samples in order to confirm the presence of veterinary drugs and pathogens.

We sincerely apologize to the readership of PLOS ONE.

